# Free will, quarantines, and moral enhancements: neuroabolitionism as an alternative to criminal law

**DOI:** 10.3389/fsoc.2024.1395986

**Published:** 2024-05-22

**Authors:** Diego Borbón

**Affiliations:** Center for Studies on Genetics and Law, Research Group on Biological Sciences and Law, Universidad Externado de Colombia, Bogotá, Colombia

**Keywords:** neurolaw, neuroethics, hard incompatibilism, prison abolition, penal abolitionism, quarantine model, neurocorrections, neurointerventions

## Abstract

This article critically navigates the complex debate surrounding free will and criminal justice, challenging traditional assumptions of moral responsibility and culpability. By exploring hard incompatibilism, which denies free will, I question the ethical justification of punitive sanctions and critically analyze the alternative models such as the public health-quarantine and nonconsensual neurobiological “moral” enhancements. These alternatives, however, introduce practical and ethical concerns. Advocating for a neuro-abolitionist perspective, through the proposition of five initial principles/debates, the article suggests a shift in integrating sociological abolitionism with insights from neuroscience. The discussion extends to the implications of hard incompatibilism and the pursuit of more humane and effective approaches to deviant behavior, ultimately calling for the abolition of punitive models and criminal law itself.

## 1 Introduction

The problem of free will has long been a central debate in philosophy, with profound implications for ethics, morality, and the law (Kane, 1996; Pereboom, 2001; Harris, 2012). Despite the multiple positions and nuances, according to Kane (2012), the free will debate revolves around compatibilism, hard incompatibilism, and libertarianism, which represent distinct perspectives on the nature of free will in relation to determinism. Compatibilism posits that free will and determinism are compatible, suggesting that individuals can possess free will even in a determined universe by acting in accordance with their desires and intentions without external impediments (Kane, 2012; McKenna and Coates, 2024). Hard incompatibilism, on the other hand, argues that free will cannot coexist with determinism or indeterminism, essentially denying the possibility of free will as traditionally conceived, due to the constraints imposed by causal determinism or the randomness associated with quantum indeterminism (Pereboom, 2001, 2009; Kane, 2012; Vihvelin, 2022). Libertarianism maintains that for free will to exist, actions must not be predetermined by prior states or events, advocating for a version of free will that requires perhaps some form of indeterminism or non-causal agency to allow for genuine choice or control over one’s actions (Kane, 2012).

This debate is particularly pertinent to the criminal justice system, where the concepts of moral responsibility and basic desert underpin the justification for punishment as traditional models of retributive criminal law rely heavily on the assumption of free will (Caruso, 2021). For that reason, hard incompatibilism denies free will, given the deterministic or truly random nature of the universe (Pereboom, 2001, 2009), raising fundamental questions on the ethical justification of punitive sanctions in criminal justice.

Moreover, one of the problems of criminal justice is that it is based on the following logic: the State holds individuals fully responsible for their actions while turning a blind eye and overlooking the true causes behind the origins of crime, preserving the anomic and marginalized social structure that was caused, or maintained, by an absent State.^1^ Then, as argued by Borbón (2022) “From this perspective, under the allegedly false narrative of free will in the penal system, the State ignores the causes of crime by holding the offender responsible and leaving the social structure intact” (p. 3). In this sense, the social failure of penal systems can be complemented with hard incompatibilism that denies the philosophical assumptions of free will, retributivism, and culpability.

In response to these philosophical challenges, scholars have proposed various alternative models to retributive justice, aiming to reconcile their proposals with the need for social order and safety. Among these, two of them stand out: the “public health-quarantine model” as advocated by Pereboom and Caruso (2018), offers a non-retributive approach that seeks to protect society from harmful behaviors without presupposing moral responsibility, based on a “right” to self-defense. From another point of view, the notion of “moral enhancement” through neurobiological interventions has emerged as a controversial proposal for preventing or treating criminal behavior (Douglas, 2008; Persson and Savulescu, 2012; De Grazia, 2014), that could serve as a futuristic alternative to incarceration and criminal sanctions. Although both alternatives, quarantine, and enhancements, have several important epistemological and practical differences, I have decided to group them as two important proposals for the future. As an example, one could think that a quarantine model could cease to be necessary if it is replaced by the possibility of intervening in criminals with neurotechnologies that “enhance” them “morally.”

It is within this contentious landscape that this article introduces a non-reductionist neurosociological abolitionist perspective, advocating for an alternative approach to criminal law that prioritizes dignity, cognitive liberty, and informed consent while critically assessing some issues with quarantine models and interventions aimed at moral enhancement. In that sense, neuroethics as “the study of the ethical, legal, and social implications of neuroscience and neurotechnology” (Muñoz, 2023), and neurolaw as an “area of interdisciplinary research on the meaning and implications of neuroscience for the law and legal practices” (Caruso, 2024, p. 4), can be useful disciplines to analyze the scope and limits of our proposal. Neuro-abolitionism, in this sense, implies building upon the foundations of sociological abolitionism, complementing this critical criminology approach with the significant contributions from neuroscience, neurolaw, and neuroethics.

## 2 Free will, quarantine models, and immoral enhancements

The implications of rejecting free will for criminal law, in our criteria, are profound.^2^ Retributive justice, which justifies punishment as a deserved response to wrongdoing, relies on the premise that individuals freely choose to commit crimes, so punishment is part of basic desert^3^ (Caruso, 2021). However, if hard incompatibilism holds, this premise is undermined, challenging the moral foundation of punitive measures. In that sense, after denying free will, the challenge “is to explain how we can adequately deal with criminal behavior without the justification provided by retributivism and basic desert” (Pereboom and Caruso, 2018, p. 203). This has led some scholars to advocate for alternative approaches to criminal justice that do not depend on notions of free will.

The quarantine model appeared in Pereboom’s (2001) *Living without Free Will* book where he drew an analogy between the “right” (of the society) to quarantine carriers of severe communicable diseases and the “right” to isolate criminally dangerous individuals, so society can justifiably detain individuals who pose a significant criminal threat (Pereboom, 2001). The analogy also implies that interventions should be no more invasive than necessary, akin to public health measures which vary in intensity based on the level of threat posed, advocating for interventions that are proportional to the actual risk posed (Pereboom, 2001). In that sense, Pereboom and Caruso (2018) concluded that their model of “incapacitation account built on the right to harm in self-defense provides the best option for justifying a policy for treatment of criminals consistent with free will skepticism” (p. 204).

This model has received objections. For example, Corrado (2016) criticizes the incapacitation model for not distinguishing between individuals who are dangerous with or without control (as captured by reasons-responsiveness), for potentially drawing too many people into the criminal justice system, and for the high costs associated with compensating those incapacitated. Moreover, the critique by Levin et al. (2023) argues that the authors misunderstand and improperly apply the concept of quarantine, failing to distinguish it accurately from isolation and overlooking significant differences in application and ethical justification between medical and criminal contexts. Additionally, they question the effectiveness of voluntary compliance strategies in crime prevention and the model’s ability to deter one-time offenders or those who commit crimes under unique circumstances. Recently Lavazza et al. (2023) focused on the unreliable tools for predicting recidivism, questioning who is truly dangerous and thus should be incapacitated. Secondly, the critics contend that the model may inadvertently encourage one-time offenders or those who do not pose a continuous threat to society to commit crimes (Lavazza et al., 2023). Further objections raised by Farina et al. (2023) concern the rights that are potentially suppressed in the quarantine model, the role of genetic justice, and the difficulty the model faces accommodating reasons-responsiveness.

On the other hand, one could enter the discussion between the interaction of a quarantine model and the possibility of moral enhancement understood as an intervention aimed at making an individual more altruistic and more oriented to justice, decreasing antimoral emotions/dispositions (Lavazza, 2017). As I had stated above, one could argue that moral enhancements could be alternatives to quarantine detentions, since the dangerousness of the subject would have been controlled. This discussion is important as some scholars have stated that, in some cases, for example, to prevent ultimate harm,^4^ moral enhancement should be compulsory (Persson and Savulescu, 2008, 2013). Bublitz (2015), in contrast, has argued that some of these proposals might lead to a Police State which he argues “are premature and dangerously off-point” (103), and that in terms of tension between rights “it seems that the punitive powers of the state cannot justify mandatory neurorehabilitation” (Bublitz, 2017, p. 29).

Fortunately, Pereboom and Caruso (2018) advocate for prioritizing rehabilitation methods that engage a criminal’s rationality, suggesting mechanical therapies as a secondary option with subject consent and prioritization of noninvasive techniques, while acknowledging the ethical considerations of more invasive methods, ultimately proposing subject choice. They frame a very interesting discussion when they mention the case of the rehabilitation of psychopaths. In this sense, they study the possibility of Deep Brain Stimulation as an alternative, assuming that even in such cases the decision to intervene remains in the hands of the subject: “[W]hen all else fails and only more invasive methods are left—for example, DBS for psychopaths—important ethical questions need to be considered and answers weighed, but leaving the final choice up to the subject is an attractive option” (Pereboom and Caruso, 2018, p. 213).

In our view, the assertions made by Pereboom and Caruso (2018) are most valid, as mandatory moral enhancements without consent would not only be immoral in a dignity-Kantian sense while infringing the most basic standards of rights and cognitive liberty but would also entail a degree of brutality (such as forcibly arresting someone to an operating room to implant a brain stimulation device) that would violate the prohibitions against cruel, inhuman, and degrading treatment or punishment.

## 3 Beyond quarantines and moral enhancements: five debates

Our analysis diverges from the quarantine model due to some epistemological, conceptual, and practical critiques, which might be a few, but end up forming substantial differences. Firstly, I agree with the academic critique that equating criminal behavior with a dangerous disease, even as a mere analogy, is problematic. This comparison, despite being acknowledged by its proponents as not solely focusing on clinical or psychiatric disorders (Pereboom and Caruso, 2018), inevitably invites academic scrutiny. The very nomenclature of an “incapacitation,” “self-defense,” and “public health-quarantine” model, limits its perceived scope, potentially obscuring the alternative approaches to conflict resolution that they support, but that remains under the shadow of the “quarantine” problem. Crime, instead of being analogical to disease, should be understood as a social construct; as most of the conflicts have *also* social and culturally situated explanations, not just neurobiological (Borbón, 2022).

The choice of “quarantine” as a descriptor for this model inadvertently invites continuous debate over its implications, despite the authors’ intentions to go beyond it. For instance, while Pereboom and Caruso (2018) discuss the promotion of restorative justice paradigms, this aspect may not be immediately apparent to readers given the model’s emphasis on detention and incapacitation. If this is the case, I fear that the public health quarantine model will continue to generate new responses and controversy, despite the author’s efforts to address each objection.

To transcend this debate, I propose to move away from the framework surrounding a quarantine model to begin new and fresh debates upon the basis of sociological penal abolitionism, complemented with neuroscience, to propose five points of discussion. Each of these five are *working* principles and I certainly welcome scholars to join this interesting debate on free will and alternatives to criminal law.

I propose as a basis of a new reflection, the sociological abolitionism of Christie (1977, 1981, 2000, 2004) and Mathiesen (2006, 2012, 2015) that criticized the illegitimacy of penal law from a sociological perspective, condemning it for being built on expanding policies of pain, with irrational and reactive criminal policies, and with a selective nature disproportionately impacting vulnerable and marginalized groups. One particularly valuable contribution that sociological penal abolitionism can offer is the understanding that crime is not a tangible entity; it is not a *being* but rather an arbitrarily designated category for conflicts (Christie, 1981). The perspectives of critical criminology are especially valuable in understanding criminal law as a contingent social construction, which is not necessary and can be deconstructed. Based on sociological abolitionism, I propose five *working* postulates for our neuro-abolitionist proposal:

### 3.1 Without free will, there can not be a culpability-based criminal law

As I see it, criminal law based on culpability and free will is problematic in light of contemporary science, which undermines the idea of free will through determinism and indeterminism, making the principle of culpability and retribution nonsensical. The reliance of criminal law on refutable metaphysical assumptions should lead to a truly scientific understanding of human behavior’s determinant factors—neurobiological and environmental— challenging the notion of culpability. Furthermore, the criminal law’s role as a social control tool and its failure to limit suffering or provide meaningful contributions to social reality, emphasizes the need for alternative approaches to justice beyond the punitive framework of criminal law. Upon this debate, I suggest a departure from traditional criminal law and culpability-based justice, advocating for future exploration of diverse alternative positions, such as what I have called *penal neuroabolitionism* (Borbón, 2021).

### 3.2 The prison system produces adverse neuropsychological effects and should be abolished over the long term

For Thomas Mathiesen (2006) “The prison does not have a defence, the prison is a fiasco in terms of its own purposes” (p. 141) as it fails to prevent crime, and does not rehabilitate, nor it brings justice. On the other hand, incarceration, as the primary sanction in contemporary criminal law, is shown to have adverse neuropsychological effects as incarceration, isolation, and degrading prison environments detrimentally affect neuropsychological well-being, leading to a significant decrease in self-control, attention, and executive functions (Meijers et al., 2015, 2018). Such environments not only potentially violate international conventions against torture but also hinder the rehabilitation process, making released prisoners less capable of leading a lawful life.

I fear that the quarantine model, upon incapacitation, could replicate the logics of prisons, or even repeat the failures of the so-called Dangerous and Severe Personality Disorder Programme which was a policy proposal in the United Kingdom for people with severe personality disorders who pose a high risk of serious offending (Wootton and Fahy, 2007). As argued by Tyrer et al. (2015) “One of the major criticisms that was made […] was that it was being used as an excuse to keep patients in detention for longer than their tariffs required. This was commonly referred to as ‘warehousing” (p. 102).

Thus, our proposal should advocate for the gradual abolition of prisons in the long term, with an immediate focus on humanizing the penal system to mitigate its harmful effects and better support rehabilitation efforts in the mid-term. A quarantine model might be supported just as an exceptional-temporal alternative in severe non-socially situated cases in which social policy and psychological mental healthcare would fail. In contrast, *consensual* therapeutic neurocorrections and interventions might, even now, be useful. As an example, the case reported by Burns and Swerdlow (2003) shows how an orbitofrontal tumor led a person to engage in sex-crimes and deviant behavior, in which, perhaps, a solely social policy or mental health approach would not work. In contrast, the consensual resection of the tumor showed a definitive solution to a clear clinical and neurological case.

### 3.3 There are more humane and effective alternatives to criminal law

The gradual abolition of criminal law can be especially useful by decriminalizing certain crimes and behaviors that are not judged to be especially serious and intolerable for society, that could be better understood as “expressions of conflicting interests” (Christie, 1981, p. 11), and be resolved with non-punitive alternatives. Problematic situations could be handled humanely and with dignity through civil, restorative justice, therapeutic approaches, and learning from alternative indigenous justice paradigms (Borbón, 2021, 2022). Christie (1977) also suggests a victim-oriented model where the affected person’s situation is carefully considered, and solutions are sought with the involvement of the person, the local community, and the state. These types of schemes can be adjusted under restorative models that do not presuppose moral responsibility and that limit the pain that the penal system causes today, while preserving proportionalities and human rights. In addition, civil law can resolve problematic situations without brutality, and restorative justice rejects the culture of punishment and vengeance, aiming to restore broken social bonds. Furthermore, therapeutic jurisprudence as proposed by Wexler (1996) is a method that views law as a potential therapeutic agent to prioritize solutions that improve the psychological well-being of those involved in a conflict, acknowledging the complexity of interactions for better lawmaking and application. What it is about, then, is to remove punitive power to the greatest possible and socially-culturally tolerable degree, with a view toward the abolition of penal systems in the long term.

### 3.4 Transdisciplinarity to understand and address human behavior

Neuroscience, criminology, and sociology are proposed as transdisciplinary tools to address human behavior, highlighting the complex interplay between social inequalities, such as extreme poverty and social exclusion, and their neuropsychological impacts. This approach highlights the importance of mental health promotion programs to mitigate disorders linked to aggression, impulsivity, and lack of empathy. This shift away from reliance on the penal system toward social programs and restorative, community-based solutions aims to reduce societal vulnerabilities and promote overall well-being, drawing inspiration from Nordic and Scandinavian countries that have successfully minimized penal measures through addressing structural social problems. I certainly agree with Pereboom and Caruso (2018) when assessing that an incompatibilist approach should “continue to endorse measures for reducing crime that aim at altering social conditions, such as improving education, increasing opportunities for fulfilling employment, and enhancing care for the mentally ill.” As argued in Borbón (2022), “The more effective social programs implemented, the less criminal law will be seen as a bitter necessity used to threaten individuals in a society” (p. 2).

### 3.5 Dignity, cognitive liberty, and consent as limits

I advocate for a conception of hard incompatibilism that respects rights, dignity, consent, and cognitive liberty, which must guide advancements in the justice system, science, and neurotechnology. Mental and neurological disorders relevant to criminology require interventions to alleviate patient and societal suffering, but these must respect informed consent, in the directions suggested by Pereboom and Caruso (2018). Thus, any legislative proposal must be grounded in the enduring principle of human dignity, alongside cognitive liberty, and consent, emphasizing the defense of our most intimate personal corner: our mind (Díaz-Soto and Borbón, 2022). Antisocial behavior can not be explained, nor addressed, exclusively as a neurobiological problem, as behaviors are also socially-culturally situated. Judicial neurocorrections should always be consensual and the last resort, prioritizing less invasive and clinically risky interventions, such as better approaches with social public policy and mental healthcare.

In general, one might argue that we do not value sentient life as inherently more or less important or morally valuable based on whether if the human or non-human animal possesses some attribute called *free will*. I argue that sentient life can be valued as worthy of respect regardless of the refutation or affirmation of *free will*.^5^ Ultimately, the intrinsic value of a sentient being who feels, thinks, loves, and suffers should not depend on a flawed metaphysical concept. Similarly, I suggest that it is relevant to differentiate free will from natural freedom or positive freedom in general. While free will would imply the principle of alternative possibilities and ultimate control, natural freedom, and positive freedom, in general, could be understood as simply the absence of external constraints, restrictions, and a basic level of volitional autonomy. This natural freedom of sentient beings may form the basis of concepts such as cognitive liberty or informed consent, which do not need to rely on the assumption of free will. As argued by Pereboom (2001) “hard incompatibilism does not imperil the reasons we have for holding that human beings have dignity, and neither does it undermine the respect that would invalidate certain forms of control and manipulation” (p. 179).

In any case, life in society will always involve a certain degree of coercion over the determined wills of human beings, so it is valid to exert social control even when people do not have free will or moral responsibility. Then, what I argue is that the degree of imposed pain used for social control should be limited, so the logics of mass incarceration, penal selectivity, and the pain caused by prisons are illegitimate and unnecessary for achieving social control.

In that direction, exceptional quarantine models must only be transitory in the medium term. In the same way, moral enhancement must respect the consent of the person. Only through incentives do I consider it valid for the state to intervene in the decision. Thus, I propose a model that goes beyond incapacitation and quarantines, for an integral adequate incompatibilist proposal that should have as its ultimate goal the abolition of criminal law, prioritizing social public policy alternatives to prevent, in advance, behaviors that harm the rights of others.

## 4 Conclusion

This article engages with the basic challenges and ethical dilemmas presented by the traditional criminal justice system’s reliance on the notion of free will and moral responsibility. Through a critical examination of hard incompatibilism, the public health-quarantine model, and neurobiological “moral” enhancements, it argues for a shift toward a transdisciplinary neuro-abolitionist perspective that respects human dignity, cognitive liberty, and informed consent. By advocating for the integration of sociological abolitionism with insights from neuroscience, neurolaw, and neuroethics, the article proposes a more humane and effective approach to address deviant behavior that transcends punitive measures. This comprehensive critique and proposition, upon five basic principles and debates, highlights the necessity of reimagining justice systems that engage with the multifaceted nature of human behavior.

Built on the foundation of hard incompatibilism, it moves beyond the issues of the quarantine model and non-consensual neurocorrections and interventions, ultimately aiming for the abolition of criminal law itself. Neuroabolitionism is not, of course, a magic formula. Unfortunately, no single proposal can address all the various possible forms of conflicts and damages on its own – a matter in which contemporary criminal law profoundly fails when facing social conflicts or mental health disorders. In that sense, moving away from the pain of the punitive paradigm is, at the very least, a step worth trying. This is an invitation to the academic community to join efforts in this direction.

## Data availability statement

The original contributions presented in the study are included in the article/supplementary material, further inquiries can be directed to the corresponding author.

## Author contributions

DB: Conceptualization, Investigation, Methodology, Resources, Validation, Visualization, Writing – original draft, Writing – review & editing.

## Footnotes

^1^In essence, criminal justice systems operate on the general principle that imputable individuals are entirely accountable for their actions. Meanwhile, these systems ignore the deeper, systemic reasons that lead to criminal behavior, such as the societal conditions of marginalization. These conditions are often a result of, or perpetuated by, a lack of effective state intervention. In this sense, State complicity could be understood in those crimes that occur in contexts of marginality, where the absence of the State makes it, in a certain way, an accomplice. As said by Brink (2021) “Structural injustice selectively compromises the state’s authority to punish crimes by the marginalized that result directly from structural injustice” (p. 224). While State complicity arguments only partially affects States legitimacy for criminal sanctions and must be distinguished on a case-by-case basis, the philosophical positions that denies free will implies a complete obstacle for culpability and retributivism.

^2^Although scholars such as Stephen J. Morse have contended that neuroscience and determinism do not undermine traditional views on free will and criminal responsibility (Morse, 2015). Recently, Morse (2023) has stated that the metaphysical discussion of incompatibilism versus compatibilism is a dialectical stalemate and a fruitless distraction to the point the author argues that “free will in the strong, metaphysical sense is not a criterion for any criminal law doctrine and is not even foundational for criminal responsibility” (par. 2). In this sense, Caruso (2021) offers substantial arguments demonstrating that penal systems do in fact base their philosophical foundations on the idea of free will.

^3^The concept of basic moral desert, as said by Pereboom (2014) implies that “for an agent to be morally responsible for an action in this sense is for it to be hers in such a way that she would deserve to be blamed if she understood that it was morally wrong, and she would deserve to praised if she understood that it was morally exemplary. The desert at issue here is basic in the sense that the agent would deserve to be blamed or praised just because she has performed the action, given an understanding of its moral statues, and not, for example, merely by virtue of consequentialist or contractualist considerations” (p. 2).

^4^Said not exactly in criminal justice, since Persson and Savulescu (2013) understand ultimate harm as to “make worthwhile life on this planet forever impossible” (p. 251).

^5^As argued by Pereboom (2001) according to Kantian ethics, human dignity is valued due to our capacities for rationality and autonomy. Practical rationality would imply the capacity for setting ends, choosing means, formulating principles and making commitments to them, while positive freedom implies a capacity to commit oneself to certain principles of conduct as rationally binding (Pereboom, 2001). However, these capacities, especially practical rationality, and positive freedom, are argued by Pereboom (2001) to remain intact under hard incompatibilism, suggesting that such philosophical position do not necessarily strip us of the attributes that confer moral dignity.
